# Unnecessary hospitalisations and polypharmacy practices in Romania: A health system evaluation for strengthening primary health care

**DOI:** 10.7189/jogh.13.04039

**Published:** 2023-05-05

**Authors:** Sophie Jullien, Irina Mateescu, Monica G Brînzac, Claudia Dobocan, Ioana Pop, Martin W Weber, Cassandra Butu, Susanne Carai

**Affiliations:** 1 World Health Organization Regional Office for Europe, Quality of care and patient safety office, Athens, Greece; 2 World Health Organization Regional Office for Europe, Child and Adolescent Health, Copenhagen, Denmark; 3 World Health Organization Romania country office, Bucharest, Romania; 4 Department of Public Health, Faculty of Political, Administrative and Communication Sciences, Babeș-Bolyai University, Cluj-Napoca, Romania; 5 Carol Davila University of Medicine, Bucharest, Romania; 6 Witten Herdecke University, Witten, Germany

## Abstract

**Background:**

Children and pregnant women usually have multiple contacts with the health care system. While most conditions can be managed by primary health care (PHC) providers, hospitalisations are nevertheless common and often unjustified. The number of hospitalizations decreased in Romania at the start of the COVID-19 pandemic. While this is likely due to the disruption of health services and public health measures established to limit the spread of COVID-19, it also suggests that a proportion of hospitalisations prior to the pandemic were unnecessary. This healthcare system evaluation in Romania quantified unnecessary and unnecessarily prolonged hospitalisations in children, pregnant women and women hospitalised for delivery, and assessed antibiotic and polypharmacy practices in these groups.

**Methods:**

We conducted the healthcare system evaluation in 10 hospitals across the country. We extracted data from medical records of patients hospitalized between 2019 and 2020. In each hospital, we randomly selected 40 medical records for each of the following groups: children 2-59 months of age, pregnant women, and women hospitalised for delivery. Clinical data were compared against WHO standards indicating a need for inpatient treatment or antibiotic therapy.

**Results:**

Among 209 children and 349 pregnant women, unnecessary hospitalisations accounted for 57.9% and 56.2% of hospitalisations, respectively. Among necessary hospitalisations, a large proportion was unnecessarily prolonged, including 44.4% (n = 32/72) in children, 23.3% (n = 34/146) in pregnant women, and 45.8% (n = 110/240) in women after delivery. The proportion of unnecessary and unnecessarily prolonged hospitalisations did not differ between the pre-pandemic, the lockdown, and the post-lockdown periods. Antibiotics were prescribed to 53.1% (n = 43/81) of children with diarrhoea, while 50.8% (n = 61/120) of women with caesarean section received an unjustified prolonged course of antibiotics. Children and women were commonly prescribed unnecessary medications.

**Conclusions:**

Findings of this evaluation should inform evidence-based decisions and actions for strengthening PHC and the healthcare system structure and improving the management of common diseases in mothers, newborns, and children. The evaluation should be repeated periodically to monitor progress.

Children under five years of age often get sick. Pregnant women require antenatal care (ANC) visits and may need medical care for pregnancy-related conditions. Most of these needs can be safely and completely managed at the primary health care (PHC) level, following standards of care [1,2]. The global strategy of Integrated Management of Childhood Illness (IMCI), launched by the World Health Organization (WHO) and the United Nations Children’s Fund (UNICEF) in the 1990s, provides standards for the management of common childhood diseases at the PHC level and for the identification of those who benefit from referral to hospital care [1]. Similarly, WHO developed standards for the management of women during pregnancy, childbirth and postpartum (Integrated Management of Pregnancy and Childbirth (IMPAC)), including early detection of complications and timely referral for hospital care [2]. The guidelines also stress the importance of the rational use of antibiotics and aim to reduce the development of antimicrobial resistance – a major global threat to the treatment of bacterial infections [3].

Observations from completed WHO missions showed that children and pregnant women with common childhood conditions were often hospitalised when they could have been safely managed in PHC [4]. Hospitalisation can lead to psychological and physical harm and can increase the financial burden on patients and the healthcare system [5-11]. Timely discharge of women after giving birth is associated with improved bonding and increased breastfeeding rates [12]. Consequently, patients should be hospitalised only when and for the time that is strictly required [13,14].

With the start of the COVID-19 pandemic, the number of children hospitalised in Romania dropped from 30 000 to 5000 between February and April 2020 [15]. This could have happened due to the disruption of health services and the establishment of public health measures to limit the spread of COVID-19, but also due to a pre-existing high number of unnecessary hospitalisations prior to the pandemic.

Obtaining data on unnecessary hospitalizations of children and pregnant women (those who could have been managed safely and entirely in PHC), and on the timely discharge of women hospitalized for delivery and their newborn babies could help with advancing our understanding of current barriers to PHC access and utilisation, identifying socioeconomic factors that influence health-care-seeking behaviours, and improving appropriate delivery and use of health services.

Additionally, observations from completed WHO missions suggested that treatment of common conditions often comprises multiple unnecessary and invasive drugs, which is neither evidence-based nor in line with international guidelines [16]. Fears associated with the COVID-19 pandemic may have exacerbated such practices, leading to the overuse of antibiotics for viral infections. Assessing the prescription of antimicrobials and other drugs in hospitalised children and pregnant women will help with understanding the magnitude of the problem and developing targeted solutions. 

By conducting this health system evaluation, we aimed to quantify unnecessary hospitalisations, unnecessarily prolonged hospitalisations, antibiotic use and polypharmacy in children, pregnant women, and women hospitalised for delivery in Romania.

## METHODS

### Study design

In December 2021, a research team travelled to 10 public hospitals across Romania to extract data from medical records (Figure S1 in the **Online Supplementary Document**). Three hospitals were maternities with no paediatric department and one was a children’s hospital without maternity care.

### Inclusion criteria

We reviewed medical records of children aged 2-59 months, pregnant women up to 37 weeks of gestation hospitalised with common diagnoses (**Table 1**), and women with term pregnancy (from 37 weeks of gestation) hospitalised for delivery between January 2019 and December 2020. We selected the diagnoses for children and pregnant women because they are important causes of disability-adjusted life years and because they were identified as one of the most common causes of hospitalisation for these population groups in Romania and globally [19,20] (National School of Public Health and Management, Romania, personal communication).

**Table 1:** Inclusion criteria for review of medical records

Population group	Inclusion criteria
Children	Hospitalised (admitted in the ward staying overnight); 2-59 mo of age; any of the following primary diagnosis (ICD-10 code): upper respiratory infection (J00-J06), pneumonia (J12-J18), acute bronchitis (J20), acute bronchiolitis (J21), other acute lower respiratory tract infection (J22), intestinal infection (diarrhoea) (A00-A09)*
Pregnant women	Hospitalised (admitted in the ward staying overnight); confirmed pregnancy up to 37 weeks of gestation; any of the following primary diagnoses (ICD-10 code): threatened premature labour up to 37 weeks of gestation (O60), threatened miscarriages up to 22 weeks of gestation (O20-O20.9), premature rupture of membranes (O42.2), mild pre-eclampsia (О14.0)
Women hospitalised for delivery	Hospitalised (admitted in the ward staying overnight) for delivery; term pregnancy: from 37 weeks of gestation

ICD-10 – International Statistical Classification of Diseases and Related Health Problems 10th revision [17], mo – months

*Young infants less than two months of age were excluded because management of such age group differs from older infants and children, as per the standards of care [18].

### Determination of necessary and unnecessarily prolonged hospitalisations

For the classification of each hospitalisation into necessary or unnecessary, the references for the standard of care were the WHO pocket book of Hospital care for children (as it is broadly used and has been utilised in similar assessments) and protocols from the Romanian Society of Obstetrics and Gynaecology, approved by the Ministry of Health of Romania and in line with WHO guidelines [18,21,22].

**Figure 1:**
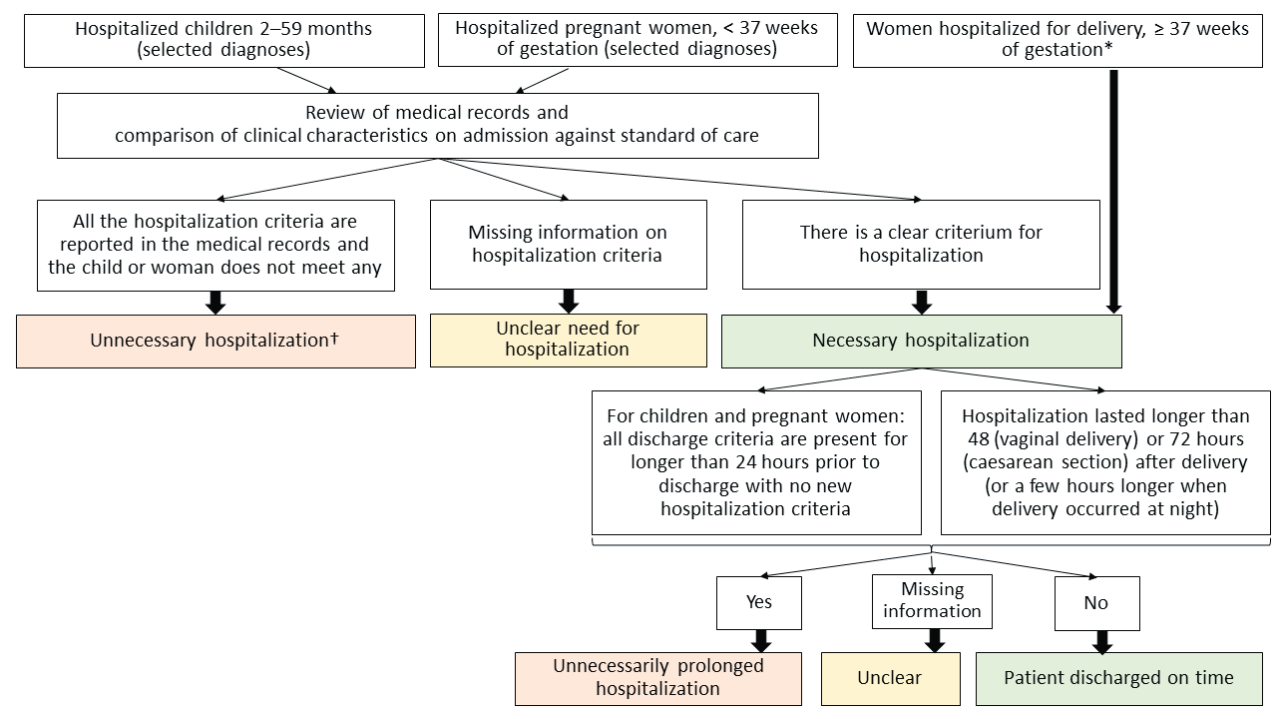
Algorithm for classification of unnecessary and unnecessarily prolonged hospitalisations. *For women hospitalised for delivery, hospitalisation was always considered necessary, as institutional delivery is a WHO standard of care. †All unnecessary hospitalisations were considered as unnecessarily prolonged.

To determine whether hospitalisations of children and pregnant women were necessary or not (**Figure 1**), we reviewed medical records for clinical characteristics present at the time of admission for the primary condition leading to hospitalisation and compared them to defined standards of care (Tables S1 and S2 in the **Online Supplementary Document**).

At the time of the reviewed deliveries (2019 and 2020), the Romanian Society of Obstetrics and Gynaecology, with the approval of the Ministry of Health of Romania, recommended that women and their babies be discharged from the hospital 48 hours after vaginal delivery and 72 hours after caesarean section if no complications had occurred [22]. Based on these criteria, we defined an unnecessarily prolonged hospitalisation for women hospitalised for delivery as hospitalisation that lasted ≥48 hours in the case of vaginal deliveries or ≥72 hours in the case of caesarean sections (**Figure 1**). We reviewed maternal medical records for any maternal, neonatal, or social condition that could have justified a longer stay; if identified, we classified the hospitalisation as prolonged with reason.

### Participant selection

In each hospital, we randomly selected 40 medical records for each of the following groups: children 2-59 months of age, pregnant women, and women hospitalised for delivery. We selected 20 files from 2019 and 20 from 2020 for each group so that they would be representative of the situation before and at the beginning of the COVID-19 pandemic. We randomly selected the medical records by picking one out of every three from the piles of paper-based records or all consecutive records matching the diagnoses of interest (depending on the way records were kept) until we reached the required number of records. We based the decision to review 40 medical records per group on feasibility and on experience from previous similar work [19].

### Data collection, management, and analysis

The data collection team reviewing the medical records and determining the unnecessary and prolonged hospitalisations included international (n = 1) and national paediatricians and midwives. We extracted the following data: general characteristics (such as date of birth or gestational week), hospitalisation and discharge dates and time, primary and secondary diagnoses, antibiotics and other drugs received prior to and during hospitalization, presence or absence of clinical signs and symptoms related to hospitalization criteria for the primary diagnosis at admission, the date when the patient met discharge criteria, and other factors possibly related with the decision of hospitalisation (such as whether the patient was referred from another health centre or came by ambulance). We recorded data into an online form (one each for children and pregnant women) or an Excel file (for women hospitalised for delivery). We analysed data with Stata (v16.0, StataCorp, Texas, USA) and used Microsoft Excel (Microsoft, Washington, USA) for the elaboration of graphs [23]. We calculated proportions for the main outcomes (unnecessary hospitalisations, unnecessarily prolonged hospitalisations, and patients receiving antibiotics and other drugs) and compared them between hospitals, pre-, during and post-lockdown related to the COVID-19 pandemic, and other subgroups of interest (age groups, time of admission, referral, use of ambulance, number of ANC visits) using the χ^2^ or Fisher’s exact test.

### Ethics

The Scientific Council of the Babeș-Bolyai University of Cluj Napoca, Romania (32/15.02.2022) approved the study.

## RESULTS

### Main characteristics

Medical records of 209 children from the seven hospitals with a paediatric department and those of 349 pregnant women and 240 women hospitalised for delivery at nine hospitals with a maternity met our inclusion criteria (Figures S2-S4 in the **Online Supplementary Document**). Their main characteristics are summarized in **Table 2** and **Table 3**. From six hospitals (data on ambulance use was not collected in one hospital), 22.9% (n = 39/170) children were brought to the hospital by ambulance, at the request of their parents or caregivers.

**Table 2:** Main characteristics of children and pregnant women

	Children (n = 209)	Pregnant women (n = 349)
Age, median (IQR)	16 (7-28) months	28 (22-33) years
Referral from PHC or other hospitals	30 (14.3%)	21 (6.0%)
Night admission (22:00-06:00)	54 (25.8%)	72 (20.6%)
Primary diagnosis	Diarrhoea (acute gastroenteritis) = 81 (38.8%)	Threatened premature labour = 186 (53.3%)
	Upper respiratory tract infection = 48 (23.0%)	Threatened miscarriage = 155 (44.4%)
	Pneumonia = 42 (20.1%)	Mild to moderate pre-eclampsia = 6 (1.7%)
	Acute bronchitis, bronchiolitis, LRTI = 38 (18.2%)	Premature rupture of membranes = 2 (0.6%)
Other diagnoses present at admission or during hospitalisation	Anaemia = 58 (27.8%)	Anaemia = 72 (20.6%)
	Metabolic acidosis = 26 (12.4%)	Urinary tract infection = 27 (7.7%)
	Malnutrition = 16 (7.7%)	Hypertension = 9 (2.6%)
	Candidiasis = 13 (6.2%)	Renal colic = 6 (1.7%)
	Rickets = 16 (7.7%)	Gestational diabetes = 5 (1.4%)
	Seizure, epilepsy = 10 (4.8%)	

IQR – interquartile range, LRTI – lower respiratory tract infection, PHC – primary health care

**Table 3:** Main characteristics of women hospitalised for delivery

	Women hospitalised for delivery (n = 240)
Age, median (IQR)	28 (22-33) years
Night admission (22:00-06:00)	64 (26.7%)
Antenatal care visits done	Complete set = 164 (68.3%)
	Inferior to recommended = 28 (11.7%)
	None = 34 (14.2%)
	Unknown = 14 (5.8%)
Type of delivery	Vaginal = 113 (47.1%)
	Caesarean section = 127 (52.9%)
Other diagnoses present at admission or during hospitalisation	Anaemia = 110 (45.8%)
	Hypogalactia = 106 (44.2%)
	Gestational diabetes = 23 (9.6%)
	Postpartum haemorrhage = 21 (8.8%)
	Perineal laceration = 20 (8.3%)

IQR – interquartile range

### Unnecessary hospitalisations

**Figure 2:**
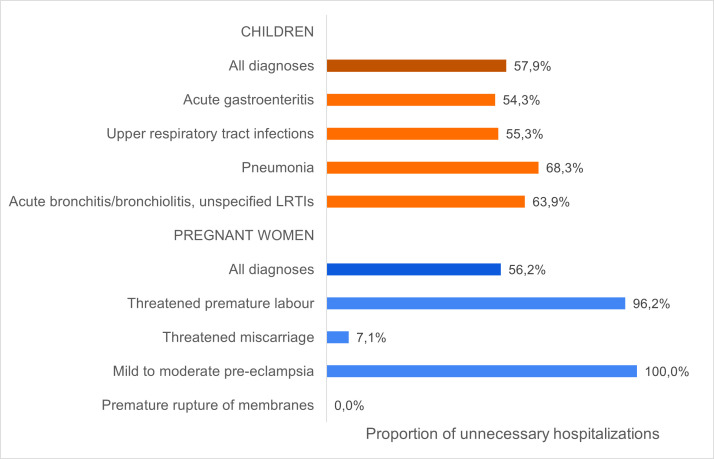
Proportion of unnecessary hospitalisations in children and pregnant women, among all diagnoses and by primary diagnosis. The total number of women used to calculate these proportions were: 349 for all diagnoses, 186 for threatened premature labour, 155 for threatened miscarriage, six for mild to moderate pre-eclampsia; and two for premature rupture of membranes.

Overall, 57.9% (n = 121/209) of children and 56.2% (n = 196/349) of pregnant women were unnecessarily hospitalised (unclear in 1.9% (n = 4/209) children)). The proportion of unnecessary hospitalisations was similar between diagnoses in children but varied in pregnant women (**Figure 2**).

In children, the proportion of unnecessary hospitalisations was similar across age groups (56.3% of infants 2-11 months vs 60.8% of children 12-59 months; *P* = 0.518), time of admission (58.7% (06:00-22:00) vs 61.1% (22:00-06:00); *P* = 0.754), and children referred to a hospital or brought in by parental decision (50.0% vs 62.4%; *P* = 0.202), but was higher among children who came by ambulance (78.4% vs 54.8%; *P* = 0.008) (Figure S5 in the **Online Supplementary Document**). Unnecessary hospitalisations in children ranged from 31.3% (n = 5/16) to 81.1% (n = 30/37) across hospitals (Figure S6 in the **Online Supplementary Document**). The hospitalisation was attributed to social circumstances in 6.7% (n = 14/209) children and 0.6% (n = 2/349) pregnant women.

### Unnecessarily prolonged hospitalisations

The median duration of hospitalisation was four days (interquartile range (IQR = 3-6)) in children and two days (IQR = 1-3) in pregnant women. It did not differ between necessary and unnecessary hospitalisations but varied across diagnoses (Figures S7-S9 in the **Online Supplementary Document**).

Among necessary hospitalisations, 44.4% (n = 32/72) were unnecessarily prolonged in children and 23.3% (n = 34/146) in pregnant women (unclear for 12 children and seven women). Findings were similar between pregnancies in teenagers (15.0%; n = 3/20) and non-teenagers (24.6%; n = 31/126) (*P* = 0.345).

The hospitalisation of 45.8% (n = 110/240) women was unnecessarily prolonged after delivery, corresponding to 57.5% (n = 65/113) vaginal deliveries and 35.4% (n = 45/127) caesarean sections, with variations across hospitals (Figure S10 in the **Online Supplementary Document**). Another 5.4% (n = 13/240) of women had a condition that justified a longer stay (i.e. a medical condition of the newborn or the mother, or social cases). The proportion of unnecessarily prolonged hospitalisation was significantly higher among women with a low number of antenatal care (ANC) visits (60.7%; n = 17/28) or none (64.7%; n = 22/34), compared with those with a complete set of ANC visits (38.4%, n = 63/164; *P* = 0.008).

### Antimicrobials

Before admission, 19.1% (n = 40/209) of children received antibiotics: 22.6% (n = 29/128) had a respiratory infection and 14.8% (n = 12/81) had diarrhoea. During hospitalisation, 66.0% (n = 138/209) of children received at least one antimicrobial and 18.2% (n = 38/209) at least two. Among children with diarrhoea, 53.1% (n = 43/81) received antibiotics.

During hospitalisation, antibiotics were prescribed to 30.1% (n = 105/349) pregnant women (with variations across hospitals; Figure S11 in the **Online Supplementary Document**), 94.5% (n = 120/127) women who gave birth by caesarean section (of whom 50.8% (n = 61) for at least two days)), and 32.7% (n = 37/113) with vaginal delivery.

### Polypharmacy

Hospitalised children and pregnant women were prescribed a median of four (IQR = 3-5) and three (IQR = 2-4) drugs, respectively, often without evidence of benefits, such as probiotics, mucolytics, and nebulized dexamethasone. Among children with acute gastroenteritis, 19.8% (n = 16/81) received trimebutine-based medicines, 11 of whom were under two years of age.

Uterotonics were prescribed to 90.0% (n = 215/240) of women after delivery for prevention or treatment of post-partum haemorrhage (PPH): oxytocin (44.6%), ergometrine (20.8%), both oxytocin and ergometrine (23.3%), and oxytocin plus ergometrine plus carbetocin (0.8%). Ergometrine was given to 45.0% (n = 108/240) of women after delivery (alone or combined with another uterotonic). PPH was documented in 8.8% (n = 21/240) of women. Additionally, 37.9% (n = 91/240) of women also received a uterotonic before delivery.

### COVID-19 pandemic

The proportions of unnecessary and unnecessarily prolonged hospitalisations were not significantly different *(P >* .05) between the pre-pandemic period, the lockdown and the period following the lockdown in each of the groups (Table S3 in the **Online Supplementary Document**). The proportion of children receiving antibiotics during hospitalisation did not vary either between the pre-pandemic period (66.4%; n = 73/110), the lockdown (69.6%; n = 16/23), and the period following the lockdown (64.5%; n = 49/76) (*P* > 0.05).

## DISCUSSION

We conducted this healthcare system evaluation in Romania following the same methodology used in Tajikistan in 2021 [19]. To the best of our knowledge, this kind of system evaluation has not previously been implemented elsewhere. It allowed quantifying in a systematic and reproducible way the proportion of patients who were unnecessarily hospitalised, i.e. who could have been managed safely and entirely from PHC services. This concept differs from potentially avoidable hospitalisations (i.e. hospitalisations which might be avoided by government policies ensuring adequate socioeconomic resources and access to high-quality housing; access to timely, appropriate and affordable PHC; and the implementation of health promotion and disease prevention strategies), and ambulatory case sensitive hospitalisations (i.e. those preventable by early and effective treatment in PHC) [24,25].

Unnecessary hospitalisations were common, accounting for 57.9% and 56.2% of hospitalisations in children and pregnant women, respectively. We observed variations in unnecessary hospitalisation rates in some sub-categories, which may allude to some of the causes behind over-hospitalisations. For example, nearly all hospitalisations for threatened premature labour were unnecessary, which could reflect a lack of protocol implementation for its management.

We found a higher proportion of unnecessary hospitalisations among children who reached the hospital by ambulance (overall 22.9% of children came by ambulance, requested by parents at no cost) and ambulance use was often not justified by the severity of the patients’ condition. These findings suggest that whether a patient comes by ambulance or by personal means may influence health workers’ decision towards hospitalisation.

While institutional delivery is encouraged as the safest option for all births [26], nearly half of the women hospitalised for delivery stayed at the hospital unnecessarily long. Unnecessarily prolonged hospitalisation was more common among women with no or a low number of ANC visits, which might be justified for awaiting results of investigations that were not performed during the pregnancy (e.g. serologies), to ensure correct management before discharge. Otherwise, prolonged hospitalisations could be partly explained by the prescription of antibiotics to babies (often unjustified), the lack of trust from the health workers to mothers in the care of their babies and the strong feeling of responsibility of the health workers, and socio-economic factors including low education and low resources (families might take advantage of hospital heating during the colder months).

We expected the proportion of unnecessary hospitalisations to decrease during the lockdown in comparison to the period prior to the COVID-19 pandemic due to lower demand (avoidance of accessing hospital care by fear to be infected) and supply (repurposing of staff and beds for patients with COVID-19) [24,27], but this was not the case. The maintained high proportion of unnecessary hospitalisations during the lockdown together with the drop in the absolute number of children hospitalised in Romania during this period [15] suggests that children with severe conditions requiring hospitalisation decreased considerably during the lockdown, probably due to drastic public health measures [24].

This evaluation also highlighted a considerable misuse of antibiotics. Children with diarrhoea do not need antibiotics (except in case of dysentery) [18], but over half of the children hospitalised with diarrhoea (no case of dysentery) received antibiotics. Women undergoing a caesarean section should receive a single dose of antibiotic for preventing peripartum infections [25], yet half received a two or three-day course of antibiotics, unjustifiably.

Moreover, children and women were commonly prescribed medication with no evidence of benefits. Oxytocin is the recommended uterotonic for both the prevention of PPH for all births [28,29] and for the treatment of PPH [30]. If unavailable or if the bleeding persists despite oxytocin, then another uterotonic (e.g. ergometrine) is recommended [28,30]. According to the reviewed medical records, 90.0% of women received a uterotonic after delivery, meaning that 10.0% either did not receive a uterotonic for prevention of PPH (and are therefore at increased risk of a preventable serious complication), or did receive it, but without the prescription being documented. However, 24.2% of women were prescribed more than one uterotonic after delivery and 45.0% received ergometrine (either for prevention or treatment of PPH). As oxytocin was available in all the hospitals and 8.8% of women were reported to have PPH, the use of ergometrine seems unjustifiably high, despite its associated increased risk of hypertension [28]. Uterotonics were also prescribed to 37.9% before delivery, unjustifiably. Likewise, one in five children with diarrhoea received trimebutine, despite no evidence of benefits and trimebutine-based medicines being associated with severe adverse effects and contraindicated in children under two years of age [31].

These findings should inform actions from stakeholders and policymakers to limit unnecessary hospitalisations and prescription of unnecessary medications. Multiple factors contribute to these challenges [32]. While quality education is surely needed, other key actions are necessary.

The first such action is improving health financing for quality of care. Allocation of public resources to the health sector in Romania has improved since 2015, but changes in behavioural practices are slower and out-of-pocket payments are still above the European Union (EU) average and dominated by outpatient pharmaceutical costs in 2019 [33]. Informal payments impact the quality of care, as they contribute to non-evidence-based prescribing practices and to supplier-induced demand for the caesarean section over a vaginal delivery and for unnecessary clinical services including testing and hospitalisations [34]. Moreover, the imbalance between PHC and inpatients remains considerable; the proportion of health spending allocated to inpatient care in 2019 was the highest among EU countries, at about 44% (although in absolute terms, the amount per person remains low compared to the EU average) [33].

The second action is strengthening PHC, which is fundamental for achieving the goal of “Health for All” [35-37]. Management of most maternal and childhood conditions at PHC with effective pathways to inform patients awaiting investigation results and continuity of care is key to reducing unnecessary and lengthy hospitalisations [32,38]. Similarly, the availability of effective social services in the community would contribute to reducing hospitalisations of healthy individuals [32,39].

The third action encompasses understanding behaviours and perceptions and identifying barriers. For example, people often consider hospital care superior to PHC (which surely lowers the threshold for hospitalisation) and often do not feel adequately cared for if no medication is prescribed by their doctor (which surely worsens the prescription of unnecessary antibiotics and other drugs) [33]. Understanding community and health workers’ practices, perceptions, and behaviours on health care, including a better understanding of factors influencing hospitalisation and polypharmacy, is key to success towards PHC and health services that are high quality for everyone and everywhere [37]. The finding of nearly half of the women with hypogalactia (defined as insufficient milk secretion to maintain exclusive breastfeeding) after delivery (**Table 3**) is likely the consequence of practices that were observed during the visit to the hospitals, including the delayed start of breastfeeding, lack of breastfeeding support, and separation of mothers and their babies after birth [40,41]. Understanding the barriers to the adoption and implementation of WHO evidence-based recommendations on the benefits of skin-to-skin and early breastfeeding should be another priority for improving the quality of health care for mothers and newborns in Romania. For all these areas of concern, we encourage qualitative research to understand behaviours and to identify perceptions of barriers and drivers experienced by the communities and by the paediatric and maternity health care workers influencing care provision. The findings will be crucial for targeted interventions, which need to be feasible and locally acceptable, to have an impact in improving the quality of paediatric and maternal care sustainably.

The fourth action is empowering patients. For example, children with wheezing requiring rapid-acting bronchodilator were directly hospitalised, independently of their immediate response to bronchodilators, as it was assumed that they cannot be managed at home and followed at PHC. Education and empowering patients and caregivers on self-management will surely contribute to reducing unnecessary and lengthy hospitalisations, as evidence shows in patients with asthma [38].

Finally, revision and enforcement of regulations for the prescriptions and dispensing of antibiotics and drugs as well as the use of ambulance service should be made a priority. Misuse of ambulance services as taxi transport should be restricted to avoid unnecessary costs to the health care system.

The limitations of this evaluation include the retrospective data collection from medical records. Information not documented in records, such as social circumstances, may lead to an overestimation of unnecessary and unnecessarily prolonged hospitalisations.

One strength of our study is that the findings are most likely applicable to the whole country (data collection in 10 hospitals with different characteristics). We randomly selected medical records and utilised a systematic, rigorous methodology, making the evaluation reproducible for data comparison and tracking progress in health systems performance with the use of highly valuable indicators including but not limited to unnecessary hospitalisations and unnecessarily prolonged hospitalisations. These two indicators may have a role in measuring the effectiveness and safety of and (indirectly) access to health care within the health systems performance assessment framework for universal health coverage [42].

## CONCLUSIONS

Our findings are useful for informing evidence-based decisions and actions from stakeholders and policymakers for strengthening PHC and improving the management of common diseases in mothers and children. This evaluation should be replicated periodically and for other areas of care so as to monitor progress.

## Additional material


Online Supplementary Document

## References

[1] World Health Organization. Integrated Management of Childhood Illness (IMCI). Geneva: World Health Organization; 2020.

[2] World Health Organization. Integrated Management of Pregnancy and Childbirth: pregnancy, childbirth, postpartum and newborn care: a guide for essential practice. Geneva: World Health Organization; 2015. 26561684

[3] World Health Organization. Antimicrobial resistance: global report on surveillance. Geneva: World Health Organization; 2014.

[4] World Health Organization. European Health Information Gateway. Available: https://gateway.euro.who.int/en. Accessed: 6 September 2021.

[5] Goslin ER Hospitalization as a life crisis for the preschool child: a critical review. J Community Health. 1978;3:321-46. 10.1007/BF01498508 730842

[6] Sheridan MS Children’s feelings about the hospital. Soc Work Health Care. 1975;1:65-70. 10.1300/J010v01n01_09 1235184

[7] Rokach A Psychological, emotional and physical experiences of hospitalized children. Clinical Case Reports and Reviews. 2016;2:10-3. 10.15761/CCRR.1000227

[8] Reyes LM Khurana R Liu F Steinback C Davenport M The impact of hospitalization on physical activity during pregnancy. J Obstet Gynaecol Can. 2021;43:766-8. 10.1016/j.jogc.2020.09.018 34099221

[9] Heaman M Psychosocial aspects of antepartum hospitalization. NAACOGS Clin Issu Perinat Womens Health Nurs. 1990;1:333-41. 2206753

[10] Loos C Julius L The client’s view of hospitalization during pregnancy. J Obstet Gynecol Neonatal Nurs. 1989;18:52-6. 10.1111/j.1552-6909.1989.tb01617.x 2926523

[11] Organisation for Economic Co-operation and Development. Tackling wasteful spending on health highlights. 2017. Available: https://www.oecd.org/els/health-systems/Tackling-Wasteful-Spending-on-Health-Highlights-revised.pdf. Accessed: 6 September 2021.

[12] Jones E Stewart F Taylor B Davis P Brown S Early postnatal discharge from hospital for healthy mothers and term infants. Cochrane Database Syst Rev. 2021;6:CD002958. 34100558 10.1002/14651858.CD002958.pub2 PMC8185906

[13] Lehner DC Sadler L Toddler developmental delays after extensive hospitalization: Primary care practitioner guidelines. Pediatr Nurs. 2015;41:236-42. 26665423

[14] World Health Organization Regional Office for Europe. Hospital care for children: quality assessment and improvement tool. Copenhagen: World Health Organization; 2015.

[15] World Health Organization Regional Office for Europe. Mitigating the impacts of COVID-19 on maternal and child health services. Copenhagen: World Health Organization; 2021.

[16] World Health Organization. Regional Office for Europe. Assessment of sexual, reproductive, maternal, newborn, child and adolescent health in the context of universal health coverage in Tajikistan. Copenhagen: World Health Organization; 2021.

[17] World Health Organization. International Statistical Classification of Diseases and Related Health Problems 10th Revision. 2016. Available: https://icd.who.int/browse10/2016/en. Accessed: 18 February 2022.

[18] World Health Organization. Pocket book of Hospital care for children. Guidelines for the management of common childhood illnesses. Geneva: World Health Organization; 2013. 24006557

[19] World Health Organization. Strengthening primary health care by avoiding unnecessary hospitalizations in Tajikistan: health systems evaluation report. Copenhagen: WHO Regional Office for Europe; 2022.

[20] GBD 2019 Diseases and Injuries Collaborators Global burden of 369 diseases and injuries in 204 countries and territories 1990-2019: a systematic analysis for the Global Burden of Disease Study 2019. Lancet. 2020;396:1204-22. 10.1016/S0140-6736(20)30925-9 33069326 PMC7567026

[21] Jullien S Mirsaidova M Hotamova S Huseynova D Rasulova G Yusupova S Unnecessary hospitalisations and polypharmacy practices in Tajikistan: a health system evaluation for strengthening primary healthcare. Arch Dis Child. 2023:archdischild-2022-324991. 10.1136/archdischild-2022-324991 36639221 PMC10313957

[22] Romanian Society of Obstetrics and Gynecology. Clinical obstetrics and gynecology reviewed guidelines from 2019. 2019. Available: https://en.sogr.ro/clinical-guidelines/. Accessed: 13 July 2022.

[23] StataCorp. Stata Statistical Software: Release 16. College Station, Texas, USA; StataCorp LLC: 2019.

[24] Arsenault C Gage A Kim MK Apoor NR Akweongo P Amponsah F COVID-19 and resilience of healthcare systems in ten countries. Nat Med. 2022;28:1314-24. 10.1038/s41591-022-01750-1 35288697 PMC9205770

[25] World Health Organization. WHO recommendation on prophylactic antibiotics for women undergoing caesarean section. Geneva: World Health Organization; 2021. 34185445

[26] World Health Organization. Pregnancy, childbirth, postpartum and newborn care: A guide for essential practice. Geneva: World Health Organization; 2015. 26561684

[27] Hategeka C Carter SE Chenge FM Katanga EN Lurton G Mayaka SMN Impact of the COVID-19 pandemic and response on the utilisation of health services in public facilities during the first wave in Kinshasa, the Democratic Republic of the Congo. BMJ Glob Health. 2021;6:e005955. 10.1136/bmjgh-2021-005955 34315776 PMC8318723

[28] World Health Organization. WHO recommendations: uterotonics for the prevention of postpartum haemorrhage. Geneva: World Health Organization; 2018. 30645062

[29] World Health Organization. WHO recommendation on routes of oxytocin administration for the prevention of postpartum haemorrhage after vaginal birth. Geneva: World Health Organization; 2020. 33252891

[30] World Health Organization. WHO recommendations for the prevention and treatment of postpartum haemorrhage. Geneva: World Health Organization; 2012. 23586122

[31] World Health Organization. WHO Pharmaceuticals Newsletter: 2017, No.5. 2017. Available: https://apps.who.int/iris/bitstream/handle/10665/272293/WPN-2017-05-eng.pdf?sequence=1&isAllowed=y. Accessed: 24 April 2023.

[32] Pope I Burn H Ismail SA Harris T McCoy D A qualitative study exploring the factors influencing admission to hospital from the emergency department. BMJ Open. 2017;7:e011543. 10.1136/bmjopen-2016-011543 28851767 PMC5577896

[33] Organization for Economic Co-operation and Development, European Observatory on Health Systems and Policies. Romania: Country Health Profile 2021, State of Health in the EU. Paris: OECD Publishing: 2021.

[34] Schaaf M Topp SM A critical interpretive synthesis of informal payments in maternal health care. Health Policy Plan. 2019;34:216-29. 10.1093/heapol/czz003 30903167 PMC6528746

[35] World Health Organization Global strategy for Health for All by the year 2000. World Health Forum. 1981;2:1-90.

[36] International Conference on Primary Health Care Declaration of Alma-Ata. WHO Chron. 1978;32:428-30. 11643481

[37] World Health Organization. Declaration of Astana: Global Conference on Primary Health Care. 2018. Available: https://apps.who.int/iris/handle/10665/328123. Accessed: 21 April 2023.

[38] Purdy S. Avoiding hospital admission: What does the research evidence say? London: The King’s Fund. 2010.

[39] NHS England. What actions could be taken to reduce emergency admissions? 2014. Available: http://www.england.nhs.uk/wp-content/uploads/2014/03/red-acsc-em-admissions.pdf. Accessed: 5 October 2022.

[40] World Health Organization. WHO recommendations on newborn health. Guidelines approved by the WHO guidelines review committee. 2017. Available: https://apps.who.int/iris/bitstream/handle/10665/259269/WHO-MCA-17.07-eng.pdf;jsessionid=CF264F9448EAE361B5BE63F6B8BD284B?sequence=1. Accessed: 10 June 2019.

[41] European foundation for care of newborn infants. European Standards of Care for Newborn Health. 2022. Available from: https://www.efcni.org/activities/projects-2/escnh/. Accessed: 17 February 2022.

[42] World Health Organization. European Observatory on Health Systems and Policies, Papanicolas I, Rajan D, Karanikolos M. et al. Health system performance assessment: a framework for policy analysis. Geneva: World Health Organization; 2022. 37023239

