# Supplementary material for: Unnecessary hospitalisations and polypharmacy practices in Romania: A health system evaluation for strengthening primary health care
Source: J Glob Health. 2023 May 5;13:04039. doi: 10.7189/jogh.13.04039 (PMC10160704; doi:10.7189/jogh.13.04039)
Supplement: Online Supplementary Document [file jogh-13-04039-s001.pdf]

Unnecessary hospitalizations and polypharmacy practices in Romania: a health systems evaluation for strengthening primary health care

Supplementary material

Figure S1. Cities where data were collected from 10 hospitals in Romania.

Figure S2. Flow chart of selection, review, and inclusion of medical records for children

Figure S3. Flow chart of selection, review, and inclusion of medical records for pregnant women

Figure S4. Flow chart of selection, review, and inclusion of medical records for women hospitalized for delivery

Figure S5. Proportion of unnecessary hospitalizations in children by age groups, time of admission, referral and use of ambulance.

Figure S6. Proportion of unnecessary hospitalizations in children, by hospital.

Figure S7. Duration of hospitalization in children and pregnant women, by primary diagnosis.

Figure S8. Duration of hospitalization by hospital in children.

Figure S9. Duration of hospitalization by hospital in pregnant women.

Figure S10. Proportion of unnecessarily prolonged hospitalizations by hospital in women hospitalized for delivery.

Figure S11. Proportion of pregnant women who were prescribed antibiotics, by hospital.

Table S1. Standards of care for assessment of unnecessary and unnecessarily prolonged hospitalizations in children.

Table S2. Standards of care for assessment of unnecessary and unnecessarily prolonged hospitalizations in pregnant women.

Table S3. Proportion of unnecessary hospitalizations and unnecessarily prolonged hospitalizations in children, pregnant women and women hospitalized for delivery, before, during (15 March to 15 May 2020) and after the lockdown.

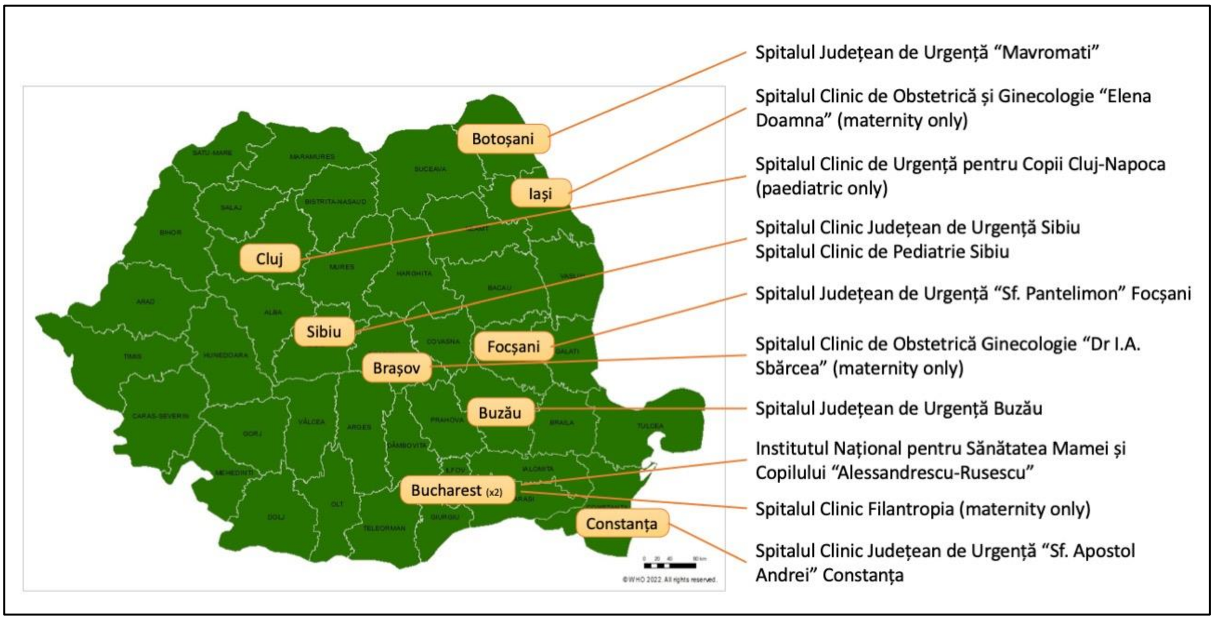

Figure S1. Cities where data were collected from 10 hospitals in Romania.

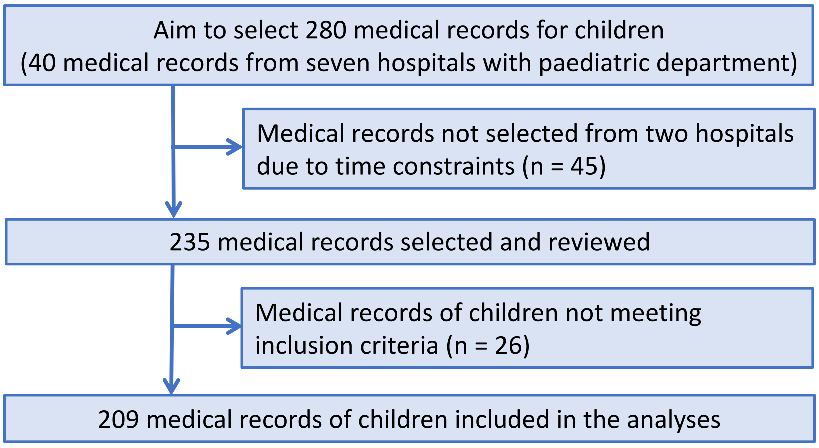

Figure S2. Flow chart of selection, review, and inclusion of medical records for children.

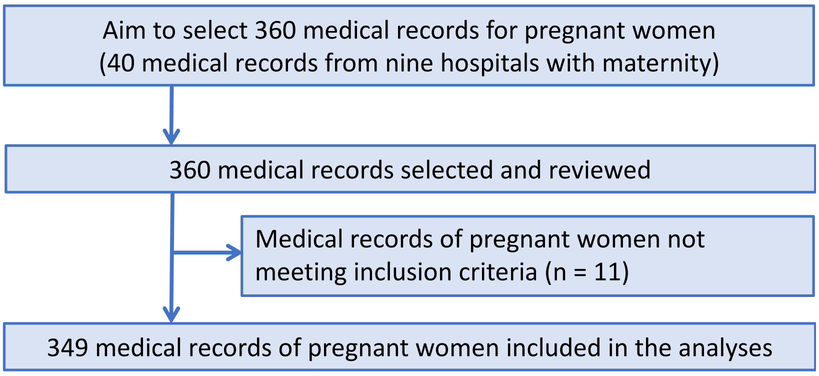

Figure S3. Flow chart of selection, review, and inclusion of medical records for pregnant women.

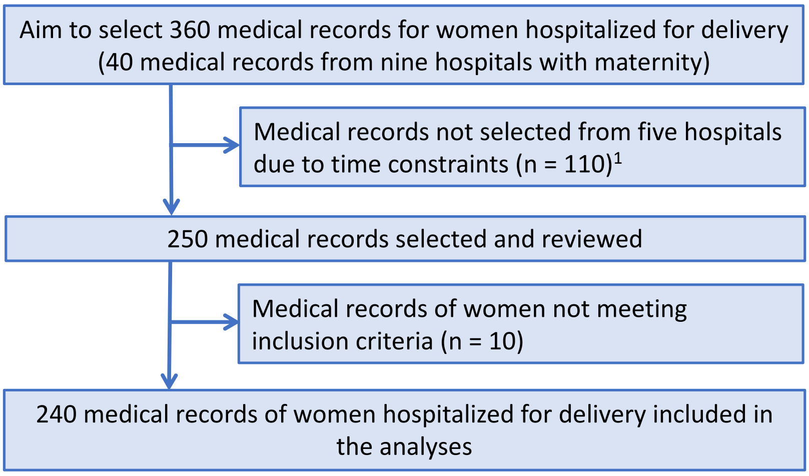

Figure S4. Flow chart of selection, review, and inclusion of medical records for women hospitalized for delivery.

[1]Medical records of pregnant women hospitalized for delivery were selected and reviewed after those for children and pregnant women. This explained the larger number of medical records that could not be selected in five hospitals due to time constraints.

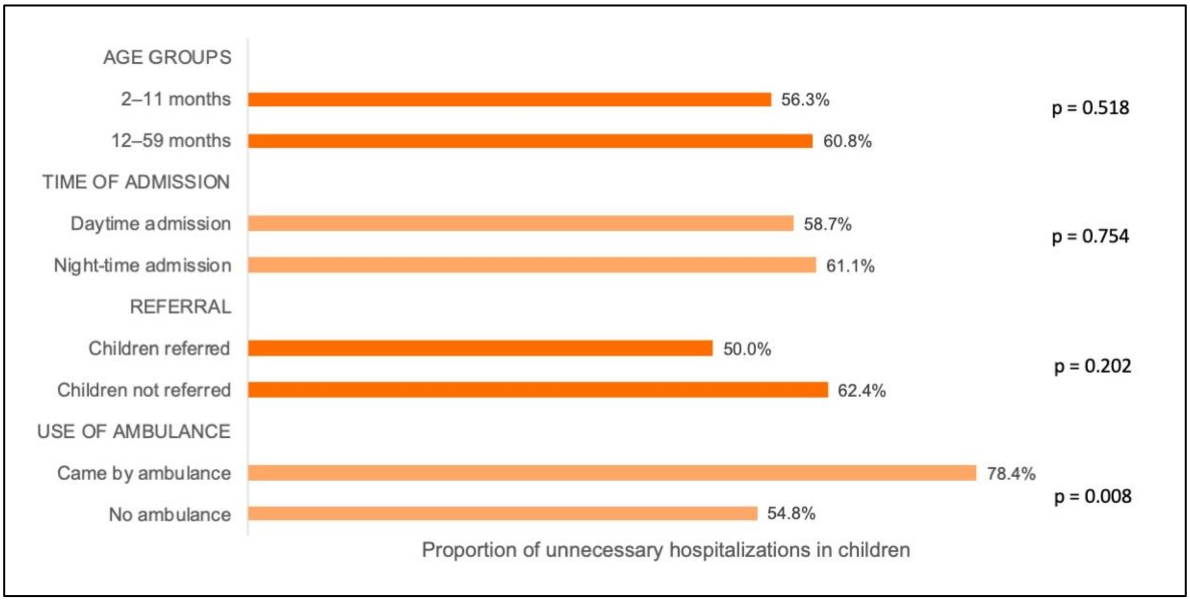

Figure S5. Proportion of unnecessary hospitalizations in children by age groups, time of admission, referral and use of ambulance.

The p-value on the right side corresponds to the comparison of proportions for each category, which was performed using the chi-square test.

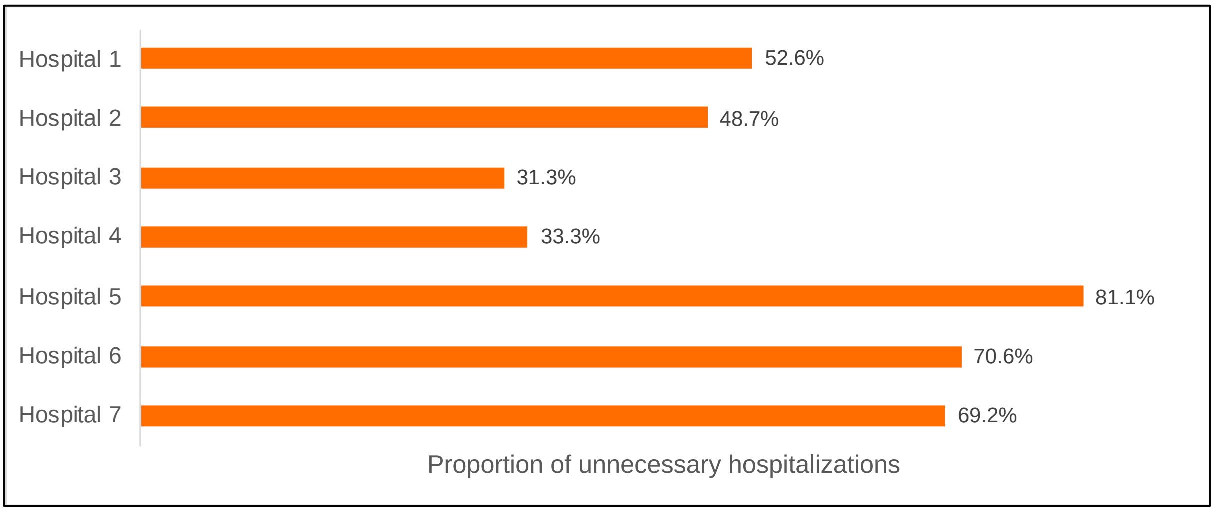

Figure S6. Proportion of unnecessary hospitalizations in children, by hospital.

Note: the hospitals in the graph were allocated random numbers to maintain anonymity of the findings.

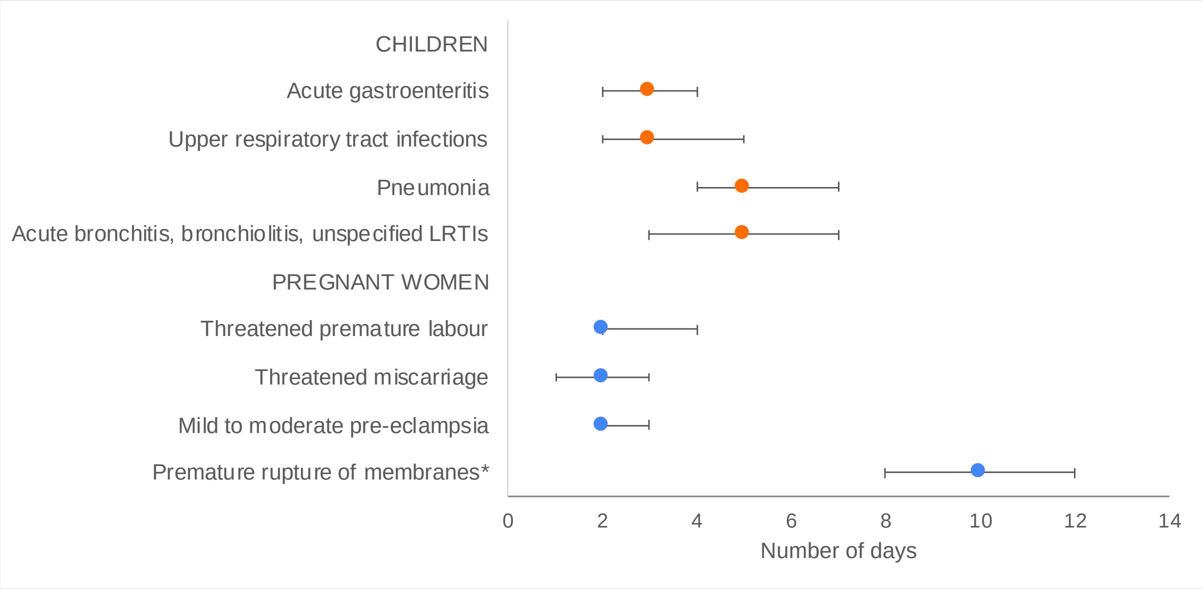

Figure S7. Duration of hospitalization in children and pregnant women, by primary diagnosis.

The orange (children) and blue (pregnant women) dots show the median duration of hospitalization, in days, and the horizontal black line the interquartile range of duration of hospitalization, for each primary diagnosis. *Note that only two pregnant women presented with premature rupture of membranes.

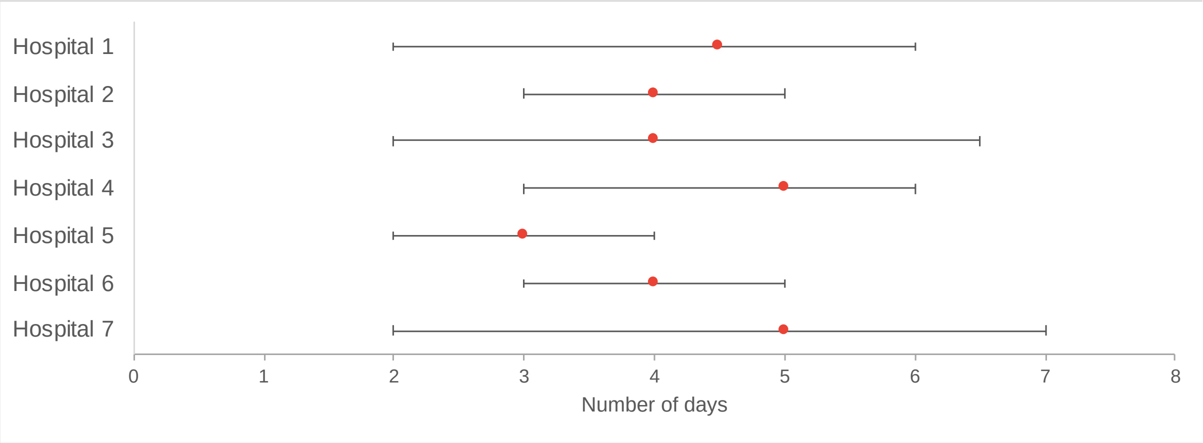

Figure S8. Duration of hospitalization by hospital in children.

The orange dots show the median duration of hospitalization, in days, and the horizontal black line the interquartile range of duration of hospitalization, for each hospital. *Note:* the hospitals in the graph were allocated random numbers to maintain anonymity of the findings.

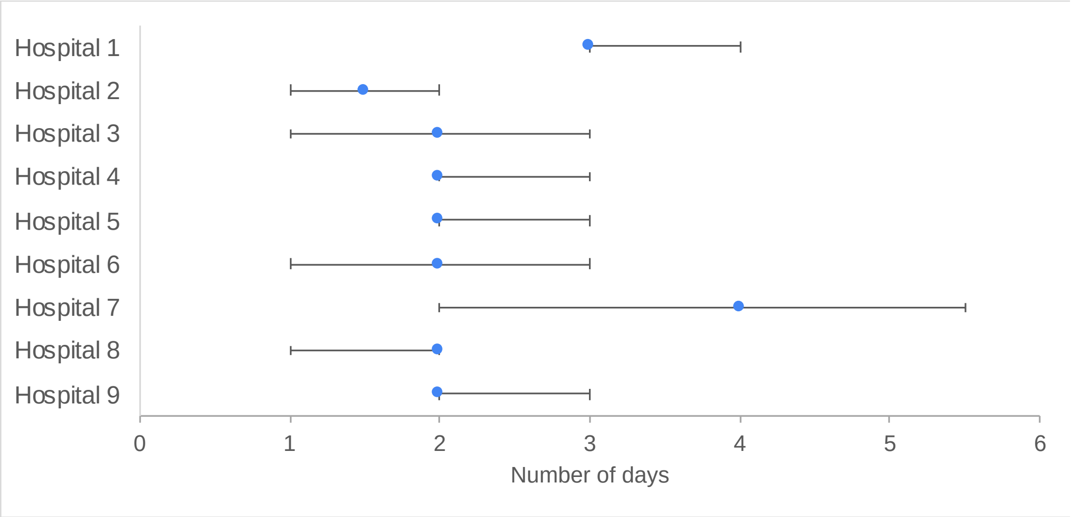

Figure S9. Duration of hospitalization by hospital in pregnant women.

The blue dots show the median duration of hospitalization, in days, and the horizontal black line the interquartile range of duration of hospitalization, for each hospital. *Note:* the hospitals in the graph were allocated random numbers to maintain anonymity of the findings.

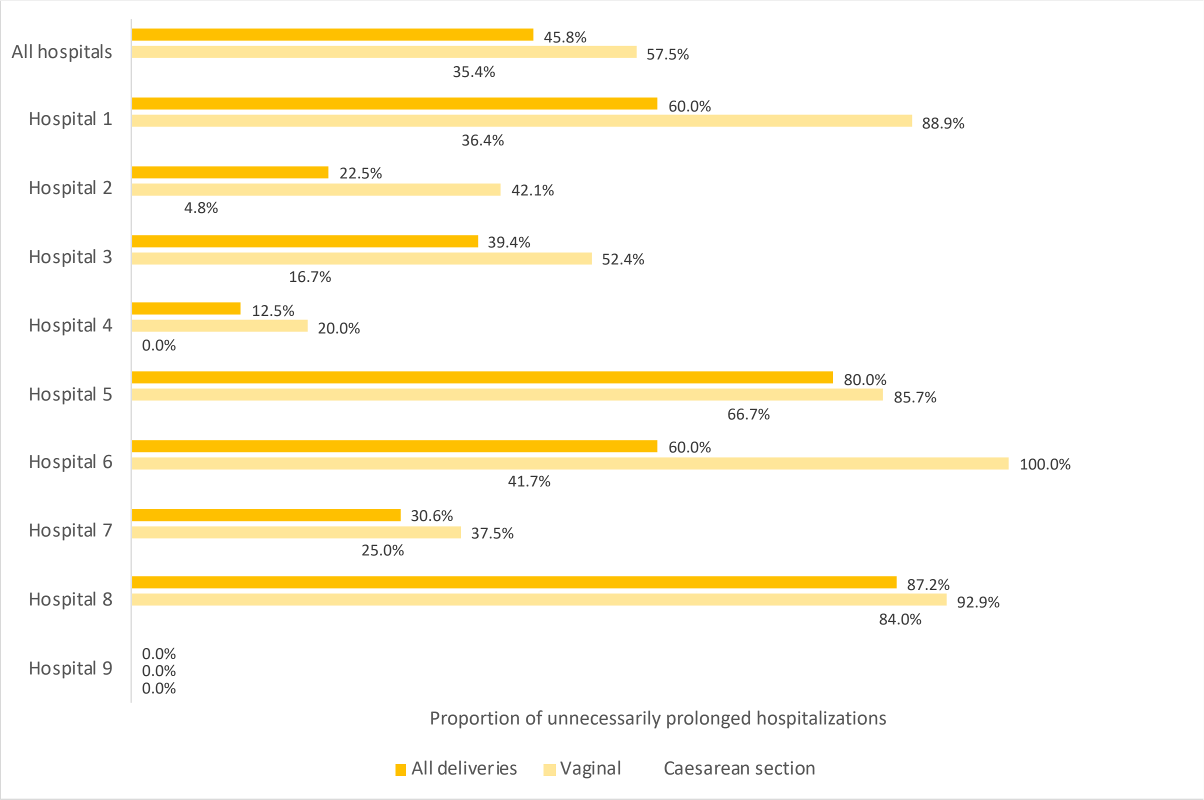

Figure S10. Proportion of unnecessarily prolonged hospitalizations by hospital in women hospitalized for delivery.

Note: the hospitals in the graph were allocated random numbers to maintain anonymity.

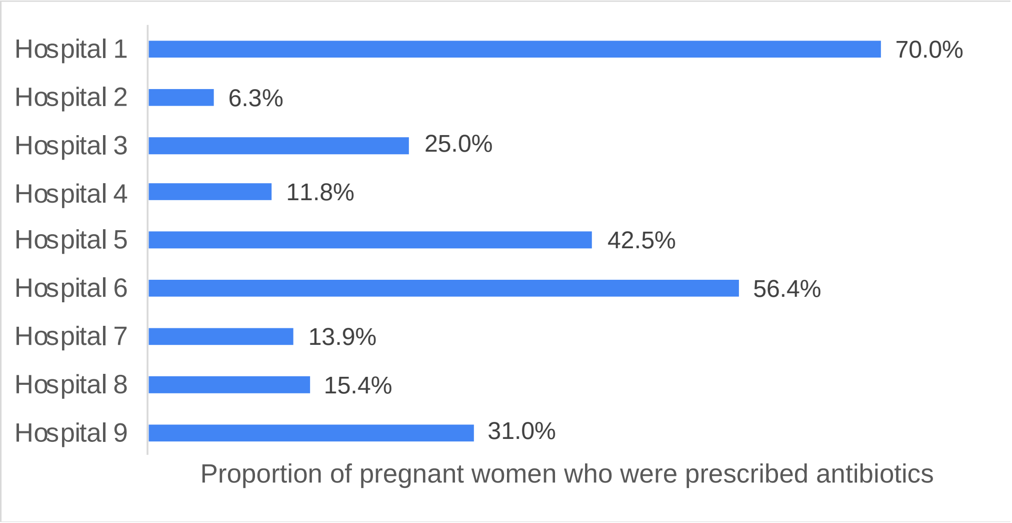

Figure S11. Proportion of pregnant women who were prescribed antibiotics, by hospital.

Note: the hospitals in the graph were allocated random numbers to maintain anonymity of the findings.

Table S1. Standards of care for assessment of unnecessary and unnecessarily prolonged hospitalizations in children.

<table>
<tr><th>Disease, condition
(ICD-10 code[1])</th><th>Hospitalization criteria[2]</th><th>Discharge criteria[2]</th></tr>
<tr><td>FOR ALL</td><td>The presentation of any general danger sign entails a hospitalization criterion by itself:
• inability to drink or breastfeed
• vomiting everything
• history of convulsions during the current illness
• lethargy, unconsciousness or convulsions.</td><td>A decision on when to discharge should be made on an individual basis, taking into consideration factors such as:
• the family's home circumstances and how much support is available to care for the child
• the staff's judgement of the likelihood that the treatment course will be completed at home or that the family will return immediately to hospital if the child's condition worsens.</td></tr>
<tr><td>Upper respiratory tract infection including common cold and croup
(J00–J06)</td><td>Severe pneumonia (see "pneumonia" below)
Severe croup, defined by any of the following:
• stridor even when the child is at rest
• rapid breathing and low chest indrawing
• oxygen saturation < 90% or central cyanosis</td><td rowspan="3">• Respiratory distress resolved
• No hypoxaemia (oxygen saturation > 90%) in room air
• No apnoea
• No stridor
• Alert
• Afebrile
• No severe dehydration
• Feeding/eating well
• Not vomiting everything
• Able to take oral medication (if needed)</td></tr>
<tr><td>Pneumonia
(J12–J18)</td><td>Severe pneumonia, defined by any of the following:
• oxygen saturation < 90% or central cyanosis
• severe respiratory distress (grunting, very severe chest indrawing)
Pneumonia not improving after three days (of oral antibiotics)</td></tr>
<tr><td>Acute bronchitis
(J20)
Acute bronchiolitis
(J21)
Unspecified acute lower respiratory tract infection
(J22)</td><td>• Oxygen saturation < 90% or central cyanosis
• Apnoea or history of apnoea
• Gasping and grunting (especially in young infants)
• Not improving after 15 minutes of rapid-acting bronchodilator (e.g. salbutamol)
- Signs of severe pneumonia (see above)
- Fast breathing: ≥ 50 breaths/minute in 2–11 months,
≥ 40 breaths/minute in 1–5 years</td></tr>
<tr><td rowspan="3">Diarrhoea, acute gastroenteritis, intestinal infectious diseases
(A00–A09)</td><td>Severe dehydration, defined as ≥ 2 of the following signs:
• lethargy or unconsciousness
• sunken eyes
• unable to drink or drinks poorly
• skin pinch goes back very slowly (≥ 2 seconds) or "reduced turgor"</td><td rowspan="3">• No severe dehydration
• Feeding/eating well
• Not vomiting everything
• Alert
• Afebrile</td></tr>
<tr><td>Severe persistent diarrhoea:
• diarrhoea lasting ≥ 14 days
• with signs of dehydration: see severe dehydration signs above, or ≥ 2 of the following signs: restlessness, irritability; sunken eyes; drinks eagerly, thirsty; skin pinch goes back slowly.</td></tr>
<tr><td>Dysentery (frequent loose stools mixed with blood) if any of the following criteria:
• < 2 months old
• severely ill children, who look lethargic, have abdominal distension and tenderness or convulsions</td></tr>
</table>

[1] ICD-10: International Statistical Classification of Diseases and Related Health Problems, 10th revision [19]
[2] Based on the WHO *pocket book of Hospital care for children* [20].

Table S2. Standards of care for assessment of unnecessary and unnecessarily prolonged hospitalizations in pregnant women.

**Disease, condition** (ICD-10 code[1])	**Hospitalization criteria[2]**	**Discharge criteria[2]**
**Threatened premature labour up to 37 weeks of gestation** (O60)	Regular contractions of the uterus lasting ≥ 30 seconds, three or more in 30 minutes **WITH** at least one of the following:<br>• rupture of foetal membranes<br>• dilatation of cervix up to 4 cm<br>• effacement of the cervix ≥ 80%	• No labour or contractions in 48 hours<br>• Full course of the respiratory distress syndrome prevention, received for at least 48 hours
**Threatened miscarriages up to 24 weeks of gestation** (O20–O20.9)	**ANY** of the following:<br>• bloody discharge or bleeding<br>• partial expulsion of products of conception<br>• cramping pains in the lower abdomen<br>• structural changes of the cervix (the cervix skips the finger)	• No bleeding in 24 hours
**Premature rupture of membranes** (between 24 and 37 weeks of gestation) (O42.2)	**ANY** of the following:<br>• pooling of fluid in the posterior vaginal fornix or clear fluid passing from the cervical canal<br>• presence of fluid on the control pad within one hour<br>• basic pH test of vaginal fluid	• Ultrasound test (amniotic fluid normal)<br>• Lack of amniotic fluid on control pad in 24 hours
**Mild to moderate pre-eclampsia** (O14.0)	• Systolic blood pressure ≥ 140 mmHg or diastolic blood pressure ≥ 90 mmHg, measured twice at an interval of at least four hours, occurring for the first time during pregnancy, after the gestational age of 20 weeks of amenorrhea, in a previously normotensive patient<br>**AND**<br>• Protein in urine ≥ 0.3 g/24 h or urinary albumin/creatinine ratio ≥ 0.3 or dipstick 1+ (if other methods are not available)	• Blood pressure below 130/80 mmHg for two days<br>• Decreased urine protein below 0.2 g/24 h or ratio < 0.2 g/l<br>• Gestation weeks ≤ 37

[1] ICD-10: International Statistical Classification of Diseases and Related Health Problems, 10th revision [19]
[2] Based on national protocols [22].

Table S3. Proportion of unnecessary hospitalizations and unnecessarily prolonged hospitalizations in children, pregnant women and women hospitalized for delivery, before, during (15 March to 15 May 2020) and after the lockdown.

		**Unnecessary hospitalizations** n/N (%)	**Unnecessarily prolonged hospitalizations** n/N (%)
**Children**	Pre-pandemic	69/108 (63.9)	12/36 (33.3)
	Lockdown	12/23 (52.2)	6/9 (66.7)
	Post-lockdown	40/74 (54.1)	14/27 (51.9)
**Pregnant women**	Pre-pandemic	98/191 (51.3)	24/87 (27.6)
	Lockdown	26/45 (57.8)	2/19 (10.5)
	Post-lockdown	72/113 (63.7)	8/40 (20.0)
**Women hospitalized for delivery**	Pre-pandemic	Not applicable	53/115 (46.1)
	Lockdown	Not applicable	19/43 (44.2)
	Post-lockdown	Not applicable	38/82 (46.3)
